# RTN4IP1 mutation and endocrine failure: clinical features and possible benefits of coenzyme Q10

**DOI:** 10.1530/EC-25-0768

**Published:** 2026-02-13

**Authors:** Lucia Digitale Selvaggio, Francesca Allosso, Martina Errico, Graziella Grande, Muhammad Yousaf, Annalaura Torella, Vincenzo Nigro, Daniela Pasquali, on behalf of Telethon Undiagnosed Diseases Program Study Group

**Affiliations:** ^1^ Department of Advanced Medical and Surgical Sciences, University of Campania Luigi Vanvitelli, Naples, Italy; ^2^ Department of Precision Medicine, University of Campania Luigi Vanvitelli, Naples, Italy; ^3^ Telethon Institute of Genetics and Medicine, Pozzuoli, Italy

**Keywords:** panhypopituitarism, *RTN4IP1*, mitochondrial disease, CoQ10, optic atrophy-10

## Abstract

*RTN4IP1* encodes a mitochondrial oxidoreductase essential for coenzyme Q biosynthesis; pathogenic variants have been reported mainly in optic neuropathy and encephalopathy. We describe a 30-year-old woman carrying three novel pathogenic *RTN4IP1* variants by exome sequencing (c.1163G>A p.Arg388Gln, c.949A>C p.Met317Leu, and c.1109T>C p.Phe370Ser), who presented with panhypopituitarism, optic nerve hypoplasia, corpus callosum agenesis, bicuspid aortic valve disease, seizures, and muscle pain, already on conventional hormone replacement. Coenzyme Q10 (CoQ10) (200 mg) was administered orally for six months; outcomes were assessed using BPI, WOMAC, TUG, LEFS, grip-strength dynamometry, SF-36, CPK, and LDH, and after six months of daily 200 mg CoQ10, the patient showed marked reductions in pain (BPI 4 → 0.8; −80%) and muscle-damage markers (CPK 254 → 110 U/L) together with gains in grip strength (+49%) and lower-extremity function (LEFS 31 → 60; +94%). SF-36 domains related to physical health showed marked gains, while emotional scores remained stable. This is the first report linking *RTN4IP1* mutations to endocrine failure and suggesting a therapeutic role for CoQ10 in mitochondrial-related endocrine disease.

## Introduction

Mitochondrial diseases are a heterogeneous multisystem disorder resulting from genetic variants affecting mitochondrial function and energy production (1, 2). *RTN4IP1*, also known as optic atrophy-10 (OPA10), is a nuclear gene encoding a mitochondrial ubiquinol oxidoreductase, an enzyme involved in the mitochondrial respiratory chain and CoQ biosynthesis (3). Biallelic mutations in *RTN4IP1* have been linked to early-onset recessive optic neuropathy, atrophy, and encephalopathy presenting with a broad spectrum of neurological features, including intellectual disability, ataxia, and seizures (3, 4, 5, 6). A study involving 12 individuals from 11 families with severe central nervous system diseases and optic atrophy identified 11 novel families with *RTN4IP1* mutations and described their association with various neurological phenotypes (4). Studies have shown that *RTN4IP1* mutations can lead to loss of the altered protein, deficits in mitochondrial respiratory complex I and IV activities, and increased susceptibility to UV light (5). The Caenorhabditis elegans ortholog, Rad8, is involved in UV light response, while silencing Rtn4ip1 in mice and zebrafish altered retinal ganglion cell dendrites, eye size, and swimming behavior *in vivo* (5). Specific mutations in *RTN4IP1* have been characterized, revealing their impact on protein function and CoQ10 biosynthesis. For example, the *R103H* mutation, linked to optic neuropathy, severely impairs the protein’s oxidoreductase activity, hindering CoQ10 production (7). *RTN4IP1*’s role is crucial in CoQ10 biosynthesis, confirming its localization in the mitochondrial matrix and function as an NAD(P)H oxidoreductase (8). This knowledge is crucial for understanding the pathogenesis of *RTN4IP1*-related disorders. By disrupting CoQ10 biosynthesis, these mutations impair mitochondrial respiration, leading to a cascade of downstream effects contributing to the observed clinical presentations. Studies on Drosophila flies with muscle-specific knockdown of the *RTN4IP1* ortholog (d*RTN4IP1*) showed promising results, significantly improving locomotor function after CoQ2 supplementation (8). These findings suggest that CoQ10 supplementation might hold promise for alleviating symptoms associated with *RTN4IP1* deficiency in humans (8). Recently, Hojabri *et al.* have reported a rare case of *COQ8A* mutation presenting with ataxia and muscle weakness, where CoQ10 supplementation (300 mg/day) led to significant functional recovery despite structural brain abnormalities (9). This emphasizes the potential for functional improvement even in complex phenotypes (9). While the neurological associations are well documented, the full clinical spectrum of *RTN4IP1* mutations, particularly regarding endocrine alterations, remains underexplored. Here, we present the first reported case of panhypopituitarism associated with three novel mutations in the *RTN4IP1* gene, expanding the known phenotypic manifestations of this rare genetic disorder. We also describe the patient’s response to CoQ10 supplementation. The continued expansion of the genetic architecture of mitochondrial diseases emphasizes the importance of understanding the role of genes such as *RTN4IP1* in disease pathogenesis. While multiple endocrinopathies are associated with primary mitochondrial diseases, including diabetes mellitus, short stature, hypogonadism, hypoadrenalism, and hypoparathyroidism, the mechanisms underlying endocrine abnormalities in mitochondrial diseases are complex and not fully understood (2). To our knowledge, this is the first report to describe an association between *RTN4IP1* mutations and endocrine alterations.

## Patient and methods

### Data collection

The patient’s clinical data were collected in an endocrine unit and analyzed at a medical genetics unit of a tertiary university clinic.

### Genetic analysis, variant validation, and interpretation

Genetic investigation was initially carried out with clinical exome sequencing (SureSelect Constitutional Panels, Agilent; sequenced on an Illumina NovaSeq 6000), with paired-end 2 × 150 bp reads with data processed through an in-house bioinformatics pipeline that filtered for non-synonymous variants and indels with a minor allele frequency < 1% while prioritizing evolutionarily conserved changes. This initial screen identified three heterozygous variants in the *RTN4IP1* gene (NM_032730.5). Whole exome sequencing (WES) was then performed using the VarGenius pipeline (GRCh37), with variant calling by GATK 3.8 and annotation using Annovar. WES confirmed the presence of three variants in *RTN4IP1*: c.1163G>A (p.Arg388Gln, exon 9, inherited from the father), c.949A>C (p.Met317Leu, exon 7, inherited from the mother), and c.1109T>C (p.Phe370Ser, exon 9, inherited from the mother); all were validated by Sanger sequencing using BigDye Terminator sequencing chemistry (Life Technologies, USA) and analyzed on an ABI 3130Xl automatic DNA sequencer (Life Technologies).

Initially, all three variants were classified as variants of uncertain significance (VUS). This was due to limited and conflicting data, as the p.Met317Leu and p.Phe370Ser variants were absent from the ClinVar and gnomAD databases, while p.Arg388Gln is listed in ClinVar as a VUS (RCV002887415.1). Furthermore, traditional in silico prediction tools, such as SIFT, PolyPhen-2, and CADD, provided inconsistent or non-definitive predictions for their effects.

To refine this classification, the variants were analyzed using AlphaMissense (10), a state-of-the-art AI prediction tool. The model classifies variants as ‘likely pathogenic’ with a score ≥0.564 or ‘likely benign’ with a score ≤0.34. The analysis yielded distinct predictions for the three variants. The p.Arg388Gln variant received a score of 0.957 (likely pathogenic), and the p.Phe370Ser variant scored 0.932 (likely pathogenic). In contrast, the p.Met317Leu variant displayed a score of 0.134 (likely benign).

These results provide additional computational evidence to reinterpret the initial findings. The data now suggest that the p.Met317Leu variant is a benign polymorphism. The patient’s phenotype is more clearly explained by a compound heterozygous state, involving the paternally inherited p.Arg388Gln and the maternally inherited p.Phe370Ser variants, both of which are predicted to be pathogenic.

### Clinical case

A 30-year-old female patient presented a complex medical history marked by panhypopituitarism, corpus callosum agenesis, bilateral hypoplasia of the optic nerves, seizures, bicuspid aortic valve stenosis-regurgitation, attention deficit disorder, central vertigo, chronic migraine, and obsessive-compulsive disorder.

The patient was born to non-consanguineous Italian parents following a physiological pregnancy and vaginal delivery. She has a brother in good health. She received surgery for a femur fracture at birth. At the age of 3–4 years, following a pediatric examination, she showed an aortic bicuspid with moderate steno-insufficiency and mild–moderate mitral regurgitation.

### Neurological alterations

From the age of 18 months, she has had episodes of loss of contact with the surrounding environment lasting a few seconds. Antiepileptic drugs were started and continued to this day, with no more episodes of loss of contact. She reported episodes of auditory hallucinations (voices and tinnitus) and obsessions with cleanliness (washing hands and changing clothes) from the age of 20. At the age of 24, she experienced an episode of involuntary movements in the lower limbs in the absence of loss of consciousness, lasting 6–7 h. Clobazam 10 mg/day was added to her therapy. A video-EEG revealed dysregulated and slowed brain electrical activity in the absence of epileptiform abnormalities. From the age of 28, the patient reports daily dizziness (objective and subjective), treated with levosulpiride. She also experiences migraine without aura, characterized by headache attacks with front-orbital localization of a pulsating character. This occurs with a frequency of about 3–4 attacks per month. She has been receiving botulinum toxin therapy for about 4–5 years with excellent clinical compensation. The patient also has attention deficit, diagnosed with ADHD.

### Endocrine features

**Figure 1:**
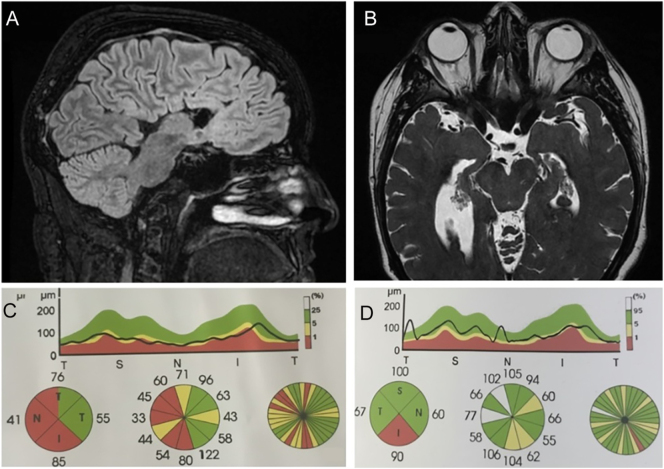
Brain MRI (A and B) and OCT examination (C and D). (A) Complete agenesis of the corpus callosum (as indicated by the arrow). (B) Bilateral hypoplasia of the optic nerves (more evident on the left) with reduced dimensions of the infraorbital and intracranial tracts (as indicated by the arrows). The RNFL OCT examination showed altered values in some sectors: left eye (C) > right eye (D).

At the age of 5, she showed a slowing of the growth rate. Diagnosis of GH deficiency was confirmed, and she started replacement therapy with somatotropin. At the age of 13, due to primary amenorrhea and the absence of secondary sexual characteristics, she was treated to induce puberty. An MRI of the brain showed complete agenesis of the corpus callosum (Fig. 1A) with secondary colpocephaly due to dysmorphism of the supratentorial ventricular system. The hypothalamic–pituitary peduncle appeared dysmorphic, due to an increase in the size of its cranial half, with ectopic neurohypophysis. Its caudal half was scarcely recognizable, giving it a truncated appearance. The pituitary gland was in place but small. She reported malaise after meals, a generalized weakness associated with nausea. Hormonal investigations revealed cortisol and ACTH values below normal. The Synacthen test confirmed cortisol deficiency; she, therefore, started a replacement treatment with hydrocortisone. She is also being treated with L-thyroxine at a substitutive dose for central hypothyroidism. The thyroid ultrasound showed a small gland and a finely inhomogeneous echo structure.

### Evaluation of optic neuropathy in *RTN4IP1* mutation

A repeat brain MRI showed bilateral hypoplasia of the optic nerves (more evident on the left) with reduced dimensions of the infraorbital and intracranial tracts (Fig. 1B). The optic chiasm was also slightly reduced in size. An eye consultation highlighted normal ocular annexes and anterior segments. Visual acuity was 8/10 natural in the right eye and 8–9/10 natural in the left eye. The intraocular pressure was 18 mmHg in both eyes. The fundus examination showed optic disks with clear margins, pinkish in color and small, and maculae within normal limits. The RNFL optical coherence tomography (OCT) examination showed altered values in some sectors (left eye > right eye) (Fig. 1C and D).

### Other medical issues

The patient had a BMI of 30.6 kg/m^2^, indicating class I obesity. She had a lipid profile characterized by hypercholesterolemia with increased LDL, treated with atorvastatin. She also had a vitamin B12 and folate deficiency. Evaluations from 2013 to date showed a slight increase in CPK and LDH values, particularly in 2023. Despite good hormone compensation, she was feeling tired and experiencing muscle pain and weakness.

### Therapeutic perspective

The patient was supplemented with a daily oral intake of CoQ10 at a dose of 200 mg before meals for 6 months.

## Results

The patient was bearing three novel sequence variants of the *RTN4IP1* gene, c.1163G>A, p.Arg388Gln in exon 9 inherited from the father and c.949A>C, p.Met317Leu in exon 7 and c.1109T>C, p.Phe370Ser in exon 9 from the mother, both apparently in good health. In our patient, the three novel variants of the *RTN4IP1* gene were associated with brain magnetic resonance imaging showing hypoplastic optic nerves (Fig. 1B) and OCT showing altered values in some sectors (left eye > right eye) (Fig. 1C and D).

Several parameters have been measured before and after 6 months of supplementation with CoQ10, including creatine phosphokinase (CPK) and LDH as markers of muscle damage. These markers decreased from 254 U/L to 110 U/L and from 310 U/L to 182, respectively, after treatment (Table 1).

**Table 1:** Parameters measured in our patient with RTN4IP1 mutation before and after 6 months of supplementation with CoQ10.

SCALE	Time 0	After 6 months	Δ%
BPI	Pain 4 (moderate)	Pain 0.8 (slight)	−80%
	Interference 5 (moderate)	Interference 0.2 (slight)	−96%
WOMAC	32/96	18/96	−43%
TUG test	14.91 s	13 s	−12%
Strength (digital dynamometer)	9.3 N	13.9 N	+49%
LEFS	31/80	60/80	+93.5%
Short Physical Performance Battery	6	5	−16%
CPK (U/L) (nv 60–190)	254	110	
LDH (U/L) (nv 120–240)	310	182	

BPI, Brief Pain Inventory; WOMAC, Western Ontario and McMaster Universities Osteoarthritis Index; TUG test, timed up and go test; LEFS, lower extremity functional scale; CPK, creatine phosphokinase; and LDH, lactate dehydrogenase.

We assessed pain, mobility, strength, and quality-of-life changes.

### Pain

The patient’s pain levels, assessed using the BPI and WOMAC scales, decreased substantially after treatment. The BPI pain score dropped from 4 (moderate) to 0.8 (slight) and the interference score from 5 (moderate) to 0.2 (slight), with a percentual variation (Δ%) indicating a decrease in pain score and interference of −80% and −96%, respectively (BPI pain score 4 → 0.8; −80%; interference score 5 → 0.2; −96%) (Table 1). The WOMAC score also improved from 32/96 to 18/96, in addition to improvements muscle-damage markers (CPK 254 U/L → 110 U/L) and gains in grip strength (+49%) and lower-extremity function (LEFS 31 → 60; +94%).

### Mobility

Several tests were used to evaluate mobility, and most showed improvements after treatment. The TUG test time decreased from 14.91 to 13 s, with a reduced time for the exercise of 12%. However, the Short Physical Performance Battery score slightly decreased from 6 to 5, suggesting that this area may not have benefited from treatment. However, the LEFS score, specifically assessing lower extremity function, showed a marked increase from 31/80 to 60/80.

### Strength

Grip strength, measured with a digital dynamometer, increased from 9.3 N to 13.9 N after CoQ10 treatment, showing a +49% increase in strength.

**Figure 2:**
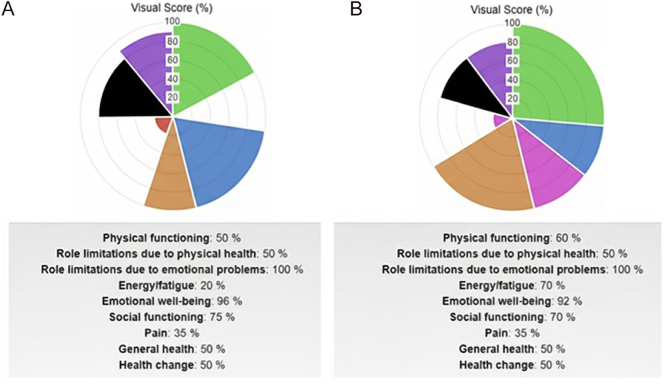
Quality of life assessed by the SF-36 questionnaire. Panels (A) and (B) show specific items and scores before and after CoQ10 supplementation for six months.

According to the SF-36 questionnaire, six months of CoQ10 supplementation led to significant improvements in several domains: physical functioning, general health, energy/fatigue, and health change, as well as a reduction in role limitations due to physical health (Fig. 2). The results of the SF-36 questionnaire excluded interference of emotional problems since emotional problems and emotional well-being were similar before and after treatment, while social functioning was clearly improved (Fig. 2).

In summary, CoQ10 treatment in this patient was associated with clear reductions in muscle suffering and pain, improvements in strength and lower extremity function, and mixed results in mobility and overall quality of life.

## Discussion

This case report presents a 30-year-old female with three novels pathogenic *RTN4IP1* gene variants, characterized by panhypopituitarism and a range of other clinical manifestations. This report is the first to describe endocrine dysfunction, particularly panhypopituitarism, in a carrier of the *RTN4IP1* mutation, broadening its known phenotypic spectrum. Previously reported cases primarily focused on optic neuropathies, encompassing isolated optic atrophy to severe early-onset encephalopathies. Another peculiarity is that our case exhibits optic nerve hypoplasia instead of optic atrophy. This observation broadens the phenotypic spectrum of *RTN4IP1* mutations, suggesting optic nerve involvement, though not always manifesting as atrophy. The patient’s intricate medical history exemplifies the multisystem nature of mitochondrial diseases (11, 12). Her presentation includes an array of neurological, endocrine, and other medical challenges, including seizures, corpus callosum agenesis, bicuspid aortic valve stenosis-regurgitation, and attention deficit disorder, associated with central vertigo, chronic migraine, and obsessive-compulsive disorder. She presented with growth delay, primary amenorrhea, cortisol deficiency, and hypothyroidism requiring hormonal replacement treatment. This diverse clinical presentation underscores the critical role of mitochondria in maintaining overall health and the far-reaching consequences of mitochondrial dysfunction. Endocrine dysfunction is a recognized feature of mitochondrial diseases, impacting various endocrine organs. The underlying mechanism is often attributed to the high energy demands of endocrine glands, making them particularly vulnerable to the effects of mitochondrial dysfunction and impaired ATP production. Diabetes mellitus is the most prevalent endocrine disorder associated with mitochondrial dysfunction (1, 2, 13, 14, 15). Notably, our case did not have diabetes but presented growth disorders and short stature, frequently observed in mitochondrial diseases. Several nuclear and mitochondrial genes have been implicated in growth disorders (14, 16, 17); likewise, hypogonadism, encompassing both hypergonadotropic and hypogonadotropic forms, has been reported (18, 19, 20). Adrenal insufficiency is less common but has been reported in various mitochondrial diseases (13, 21). While thyroid dysfunction has been reported in some cases, its prevalence in large cohorts of mitochondrial patients appears to be like that of the general population (22, 23).

Endocrine dysfunction, in particular growth hormone deficit, is an increasingly recognized feature of mitochondrial diseases. In the case series by Charif *et al.*, growth retardation was identified as a common symptom in patients with *RTN4IP1* mutations (4). Our case further expands this endocrine landscape by documenting complete panhypopituitarism associated with novel *RTN4IP1* variants.

However, our patient’s neuroimaging findings, including pituitary hypoplasia and corpus callosum agenesis, in the context of a septo-optic dysplasia spectrum, could provide a more direct explanation for the endocrine phenotype than the impaired energy metabolism.

*RTN4IP1* encodes a mitochondrial protein essential for the biosynthesis of CoQ. CoQ is a vital component of the mitochondrial respiratory chain, crucial for cellular energy production and antioxidant defense, and is highly conserved across species. The observed positive clinical response to CoQ10 supplementation, evidenced by improvements in pain, strength, mobility, and functional capacity, strongly supports the hypothesis that CoQ deficiency plays a significant role in the pathogenesis of *RTN4IP1*-related disorders. These findings are consistent with prior observations in fruit fly models of *RTN4IP1* deficiency, where CoQ supplements were shown to mitigate locomotor defects.

Our patients showed a reduction in CPK levels from 254 to 110 U/L. As documented by large population-based studies, for a Caucasian woman with the patient’s characteristics, the baseline levels of 254 U/L exceed the expected physiological range, which typically remains below 150–180 U/L, reflecting a state of chronic muscle suffering (24, 25). Thus, the decrease to 110 U/L represents a normalization of muscle enzymes and a reduction in muscle suffering that correlates with the observed clinical and functional improvements.

Although the administered dose of CoQ10 (200 mg/day, 2 mg/kg/day) is lower than the dose typically used in primary CoQ10 deficiencies, it was sufficient to elicit a functional and clinical improvement in our case, since our patient does not carry a defect in the CoQ biosynthetic pathway, but rather a novel mutation in *RTN4IP1*. While *RTN4IP1* is essential for CoQ biosynthesis, the precise metabolic requirements for supplementation in *RTN4IP1*-related disorders are not yet fully established in clinical practice. Despite the distinct molecular bases, our results are consistent with recent reports on other forms of CoQ10 deficiencies as shown by Hojabri *et al.*, who described a patient with *COQ8A* mutation exhibiting significant clinical recovery after CoQ10 supplementation (300 mg/day), with a notable improvement in muscle strength and the disappearance of neurological symptoms such as head tremors (9). Moreover, the lack of baseline CoQ10 levels and longitudinal data on lactate and organic acids represents a limitation of this study, as these markers could have provided further biochemical support to the clinical findings observed.

Our case report makes a significant contribution to our understanding of the clinical and genetic spectrum of *RTN4IP1*-related disorders and strongly emphasizes the need to consider mitochondrial diseases in the differential diagnosis of patients presenting with multiple endocrinopathies, particularly when accompanied by atypical features. The limitation of this observation is that this is a single-case study, and the observed effects cannot be definitively attributed to CoQ10 treatment alone. Controlled studies with larger patient groups are needed to confirm these findings and establish the efficacy of CoQ10 for treating conditions caused by *RTN4IP1* mutations.

## Declaration of interest

The authors declare that there is no conflict of interest that could be perceived as prejudicing the impartiality of the work reported.

## Funding

This work was supported by EU Horizon 2020 research and innovation program (Grant Agreement Number 779257), Fondazione Telethon (Grant No. GSP15001), the Ministry of Health (PNRR-MR1-2022-12376412), and the Ministry of University and Research (MUR; PRIN2022 code 2022L4F87B to VN). This work was also supported by the Ministry of University and Research (MUR) – PRIN: PROGETTI DI RICERCA DI RILEVANTE INTERESSE NAZIONALE – Bando 2022 Prot. 2022NCL5JE to DP.

## Data availability

The data of this study are available from the corresponding author upon reasonable request.

## Ethic dissemination

The patient’s clinical data were collected from the Program Unit of Rare Endocrine Diseases, AOU Vanvitelli, in collaboration with the Telethon Undiagnosed Disease Program (TUDP) and the medical genetics laboratory, Department of Precision Medicine, University of Campania ‘Luigi Vanvitelli’, Naples, Italy. Written informed consent for genetic analysis and for the publication of all identifiable clinical and molecular information was obtained from the patient. The study was approved by Campania Territorial Ethics Committee 2, Protocol Number: 0018726 of 04/07/2024.
